# Supplementary figures and images for: High DAPK1 Expression Promotes Tumor Metastasis of Gastric Cancer
Source: Biology (Basel). 2022 Oct 11;11(10):1488. doi: 10.3390/biology11101488 (PMC9598723; doi:10.3390/biology11101488)

Figure 4A

DAPK1

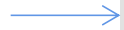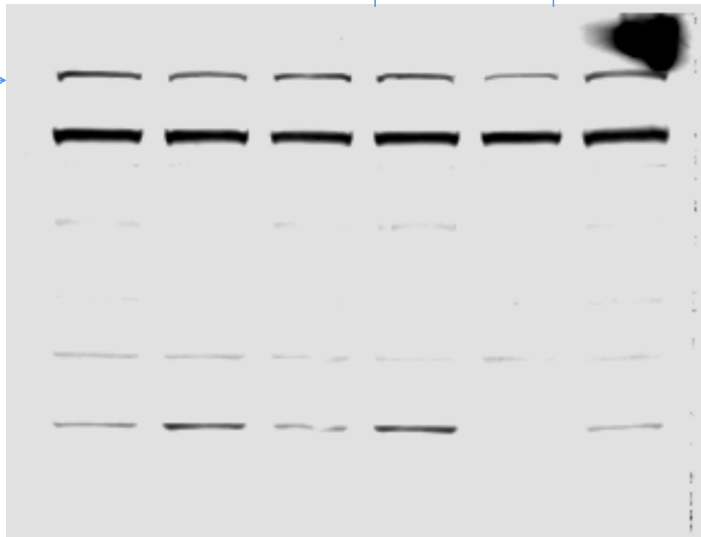

GAPDH

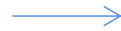

Figure 4A

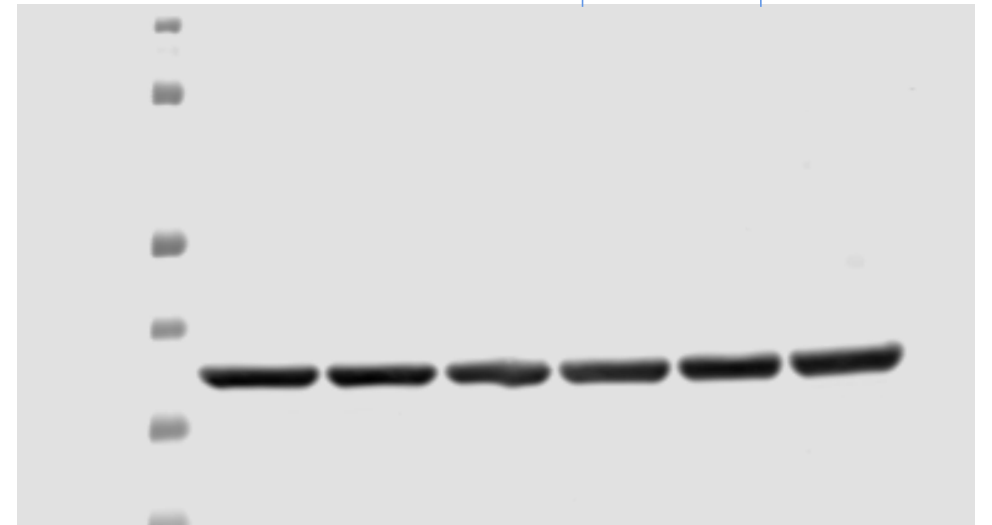

Supplement: Supplementary file 1 [file biology-11-01488-s001.zip › biology-1906788-supplementary materials/Figure S1 Original Images of Western Blot of Figure 4A.pdf]

Figure 5A

HA-DAPK1

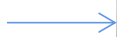

GAPDH

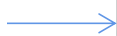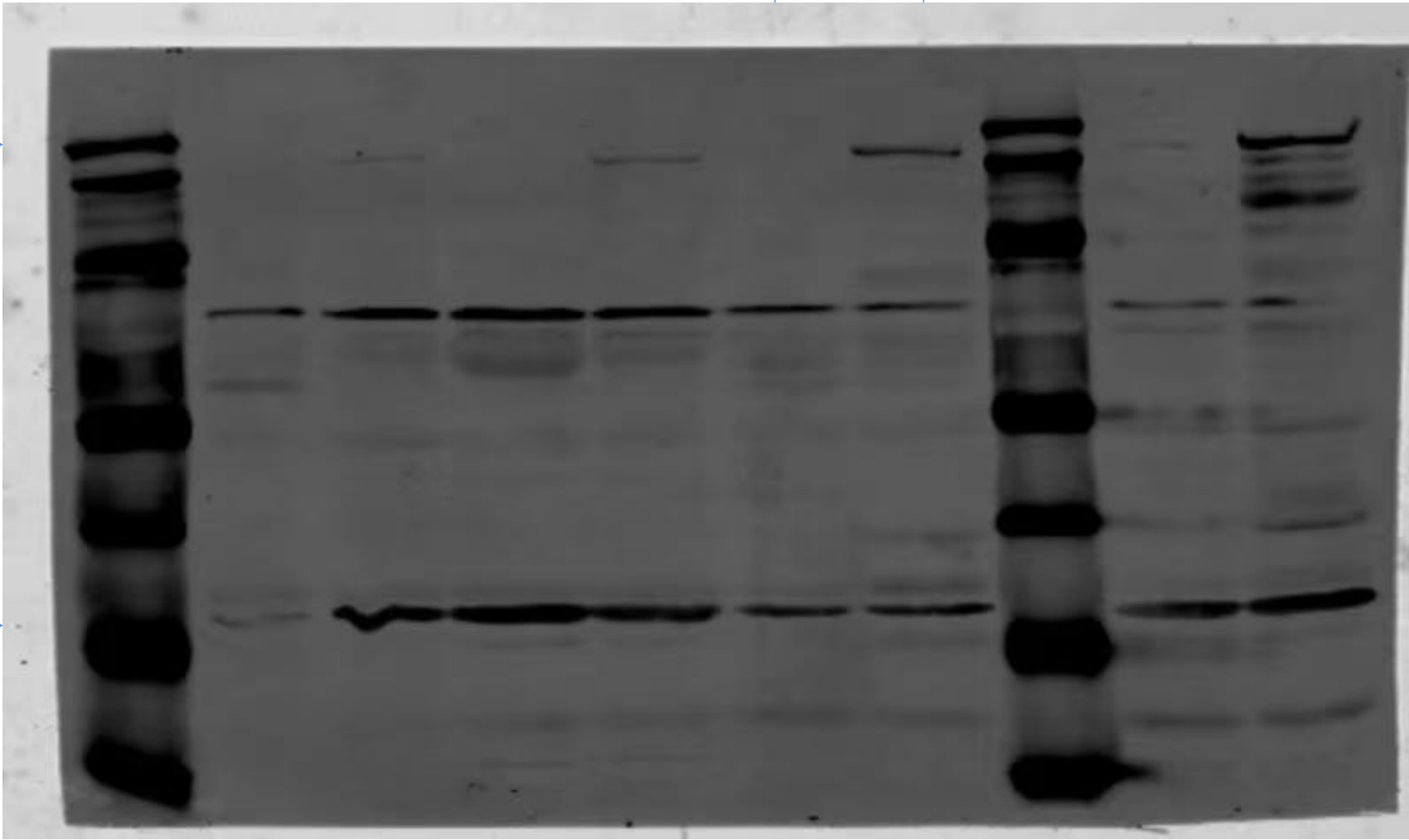

Supplement: Supplementary file 1 [file biology-11-01488-s001.zip › biology-1906788-supplementary materials/Figure S2 Original Images of Western Blot of Figure 5A.pdf]
